# Testing the optimal defense hypothesis in nature: Variation for glucosinolate profiles within plants

**DOI:** 10.1371/journal.pone.0180971

**Published:** 2017-07-21

**Authors:** Rose A. Keith, Thomas Mitchell-Olds

**Affiliations:** 1 University Program in Genetics and Genomics, Duke University, Durham, North Carolina, United States of America; 2 Biology Department, Duke University, Durham, North Carolina, United States of America; Rutgers The State University of New Jersey, UNITED STATES

## Abstract

Plants employ highly variable chemical defenses against a broad community of herbivores, which vary in their susceptibilities to specific compounds. Variation in chemical defenses within the plant has been found in many species; the ecological and evolutionary influences on this variation, however, are less well-understood. One central theory describing the allocation of defenses in the plant is the Optimal Defense Hypothesis (ODH), which predicts that defenses will be concentrated in tissues that are of high fitness value to the plant. Although the ODH has been repeatedly supported within vegetative tissues, few studies have compared vegetative and reproductive tissues, and the results have not been conclusive. We quantified variation in glucosinolate profile and tissue value between vegetative and reproductive tissues in *Boechera stricta*, a close relative of *Arabidopsis*. *B*. *stricta* manufactures glucosinolates, a set of defensive compounds that vary genetically and are straightforward to quantify. Genetic diversity in glucosinolate profile has been previously demonstrated to be important to both herbivory and fitness in *B*. *stricta*; however, the importance of glucosinolate variation among tissues has not. Here, we investigate whether allocation of glucosinolates within the plant is consistent with the ODH. We used both clipping experiments on endogenous plants and ambient herbivory in a large-scale transplant experiment at three sites to quantify fitness effects of loss of rosette leaves, cauline leaves, and flowers and fruits. We measured glucosinolate concentration in leaves and fruits in the transplant experiment, and asked whether more valuable tissues were more defended. We also investigated within-plant variation in other aspects of the glucosinolate profile. Our results indicated that damage to fruits had a significantly larger effect on overall fitness than damage to leaves, and that fruits had much higher concentrations of glucosinolates, supporting the ODH. This is, to the best of our knowledge, the first study to explicitly compare both tissue value and chemical defense concentrations between vegetative and reproductive tissues under natural conditions.

## Introduction

Plants manufacture a wide range of defenses against herbivores; variation in these defenses is common, both among individuals and within a single plant. Variation in physical defenses among plant tissues is often easily observable; many chemical defenses vary among tissues as well [1]. Studies of this variation have given rise to a number of general hypotheses regarding the evolution and allocation of defenses. The Optimal Defense Hypothesis (ODH) is one of the primary theories addressing the distribution of defenses within a plant. The ODH, which very broadly states that defenses will be allocated in any way that is adaptive to the plant, may be extended into a set of specific, testable predictions for defense allocation [2–4]. Within the plant, the ODH predicts that defenses will be concentrated in tissues that are at higher risk of herbivory and/or tissues that are more valuable; that is, tissues which if lost to herbivores cause greater loss of plant fitness [2–4].

Conventional wisdom holds that, within this framework, younger leaves are more valuable than older, and that the fitness effects of losing reproductive tissues are greater than losing vegetative tissues [5]. Most of the studies that have explicitly examined tissue value of young and old leaves support these assumptions [1, 6–8]. Less work has been done, however, to test the assumption that reproductive tissues are both more valuable than leaves and more heavily defended. While the relative importance of reproductive tissues compared to leaves may seem obvious, the question deserves investigation. If decrease in productivity from loss of leaf tissue reduces flower number or fruit set, it may cost the plant more than relatively late-stage fruit damage. Some evidence, both direct and circumstantial, also suggests that reproductive tissues, particularly flowers, may not be more valuable than young leaves. Comparisons between leaves and flowers are equivocal on which tissue is more heavily defended [1, 9, 10]; one potential explanation for this deviation from the expectation of higher defenses in reproductive tissues is that flowers are not in fact more valuable than leaves. When directly quantified, the fitness effects of loss of flowers have been found to be less than the effects of losing leaf tissue [10]. Some plants are highly tolerant of loss of floral tissue, including entire flowers; some even over-compensate, producing more flowers or fruits following floral loss [11, 12]. Multiple mechanisms of tolerance to floral loss have been documented [12, 13]. Loss of flowers does not necessarily leading to a corresponding loss of fruits or seeds. In *Boechera stricta*, the experimental system used here, we often observe buds that do not develop into flowers of fruits (personal observation); thus, we hypothesized that if loss of flowers or young fruits induced development of more buds, plants might compensate for tissue loss. Generally, the question of whether reproductive tissues, especially flowers and immature fruits, have higher fitness value than leaves is still unresolved. As such, an explicit comparison of the value of leaf and reproductive tissue under natural conditions is necessary to test the assumption of higher values for reproductive tissues. To the best of our knowledge, no such study comparing both fitness values and defense allocation in leaves and fruits under natural field conditions has previously been completed. Here, we fill this gap by explicitly comparing the value of young leaves and immature fruits, using both clipping experiments and ambient herbivory, and ask whether the more valuable tissues are more heavily defended. For this study, we used *B*. *stricta*, which manufactures glucosinolates, metabolically active secondary compounds that are involved in defense against herbivores in many crucifers [14].

The ODH makes predictions about the total concentration of defenses found in each tissue [4, 15]. In some species, the types of chemical defenses also vary among tissues [16–18]. Such variation may be due to different selective regimes exercised on flowers and fruits by animal pollinators and/or dispersers, if the plant relies on either [19, 20]. Variation between tissues is found even in species that do not rely on animals, however, such as the extensive variation in glucosinolate types among leaves, fruits and seeds in *Arabidopsis thaliana* [17]. Whether this variation affects herbivore damage is unclear. We investigated variation between leaves and young fruits for not only concentration of defenses, as needed to test the ODH, but also other types of variation. *B*. *stricta* manufactures up to four distinct types of aliphatic glucosinolates, chemical defenses common among crucifers. Site-specific effects of chemical defenses on herbivore damage have been demonstrated in leaves [21], but little work has addressed variation in the reproductive tissues in this species. As *B*. *stricta* is predominantly selfing and does not use animals to disperse seeds, variation between vegetative and reproductive tissues is not likely to be driven by mutualistic interactions. Thus, in addition to testing the ODH, we ask whether chemical variation between leaves and fruits is adaptive and driven by herbivores, by testing whether variation in the relative proportions of each glucosinolate (hereafter, “glucosinolate profile”) affects herbivory on stem leaves and fruits. Here, glucosinolate profile is described using three ratios. One of these, the proportion of glucosinolates derived from branched chain amino acids (PropBC), has been previously shown to affect herbivory and fitness under natural conditions [21], making it of particular interest. Given the evidence that the relative ratios of specific glucosinolate compounds can affect herbivory, we also investigated other aspects of glucosinolate profile; the other two proportions used (described in the Methods) were chosen to capture the remaining variation in glucosinolate profile.

## Methods

### Study system

**Fig 1:**
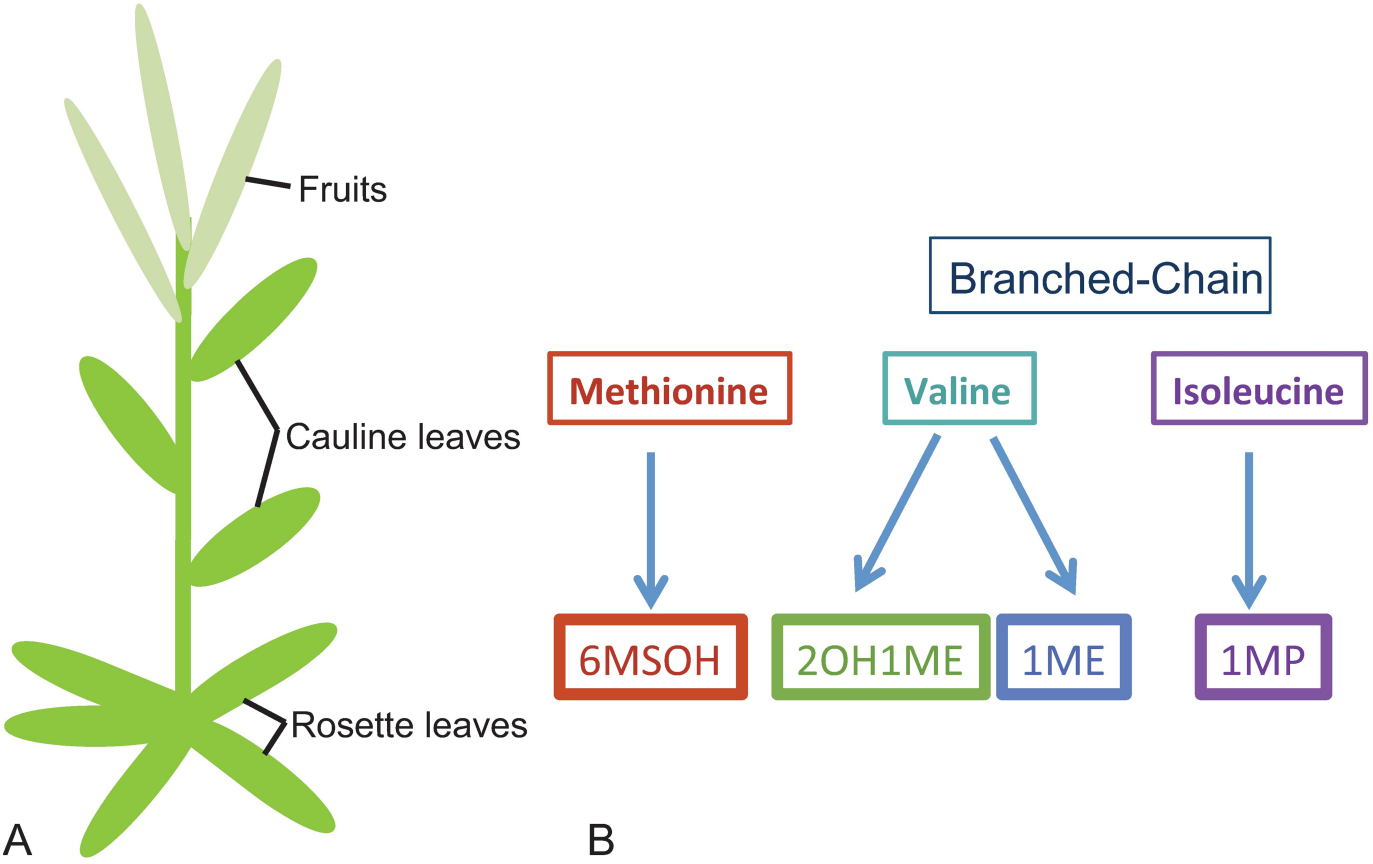
Tissue types and glucosinolate variation in *B*. *stricta*. (A) Tissue types in *B*. *stricta*. Cauline leaves and fruits are produced within one growing season, following bolting. (B) Aliphatic glucosinolates of *B*. *stricta*. Each glucosinolate type is derived from methionine or one of two branched-chain amino acids, valine or isoleucine. Two types are derived from valine.

We performed this field experiment using *Boechera stricta*, a short-lived perennial crucifer that is a model species for ecological and evolutionary genetics [21–23]. *B*. *stricta* is predominantly self-pollinating, and does not rely on animals to disperse seeds [22]. Closely related to *Arabidopsis*, *B*. *stricta* grows in montane environments in western North America. Some of these sites have been relatively undisturbed for approximately 3,000 years [24], enabling the development of long-term local adaptation and evolutionary relationships. During a reproductive season, plants advance from a vegetative rosette stage to a reproductive one, growing a stalk with cauline leaves, flowers and siliques (Fig 1A). In the field, endogenous and transplanted *B*. *stricta* plants are attacked by a diverse community of herbivores that includes species from Lepidoptera, Diptera, Coleoptera, Orthoptera, Hemiptera, and more (personal observation). Like many other crucifers, *B*. *stricta* uses a glucosinolate-myrosinase system as a chemical defense [25]. Diversity in glucosinolate structure arises from different amino acid precursors and variation in secondary modifications; *B*. *stricta* manufactures four types of aliphatic glucosinolates, derived from methionine, valine, or isoleucine (Fig 1B). The methionine-derived glucosinolate is 6-methylsulfinyl hexyl (6MSOH); from the branch chain amino acids, isoleucine gives rise to 1-methyl propyl (1MP), and valine gives rise to both 1-methyl ethyl (1ME) and 2-hydroxy 1-methylethyl (2OH1ME). Testing the ODH requires that defenses must be highly similar between tissues, in order for comparisons to be accurately made [15]. Given the close biochemical relationships among the glucosinolates found in *B*. *stricta*, and the fact that all glucosinolates can be found in all tissues, just in different proportions, we believe that this assumption is appropriate in this experiment.

The region of central Idaho where this work took place is a contact zone for two subspecies of *B*. *stricta* [26]. The two subspecies, East and West, are genetically and morphologically differentiated and are generally found in divergent environments [23, 26, 27]. Western genotypes are generally found in wetter riparian areas, while Eastern genotypes are often found on higher elevation slopes with lower water availability [26]. These subspecies have experienced divergent selection on phenology and morphology traits consistent with adaptation to these environments [27].

### Transplant experiment

Sites: Plants were grown in three gardens in central Idaho. The area where these sites are located has been relatively undisturbed for approximately 3000 years [24]. The sites differ ecologically. SIL, at 1830 meters elevation, is a shady riparian site, with dense vegetation. JAM, at 2680 meters elevation, is a high montane meadow with limited vegetation. MAH, at 2530 meters, is a sagebrush meadow. All sites occur on public Forest Service land, and were used under a Special Use Permit from the Salmon-Challis National Forest Service, and from the US Forest Service Northern and Intermountain Regions. At all three sites, plants were grown in gardens with fences to deter cattle.

Seed collection and plant growth: Seeds from naturally inbred individuals were collected at 24 ecologically diverse sites in central Idaho and one site in Colorado (the Colorado genotype was excluded from all later analyses) [26]. These collections were split equally between the East and West subspecies, and had been used for previous work on the ecological and genetic differentiation between the subspecies [26, 27]. Seeds were grown in the greenhouse for a generation to minimize maternal effects before being used for experiments.

Seeds for experimental plants were germinated in petri dishes. Germinating seeds were kept in the dark at room temperature for 3 days, and then transferred to 14 hour days at 20 degrees. At one week they were transplanted into 2.5 cm by 12 cm plastic conetainers (Stuewe and Sons, Inc.), and grown in the Duke University greenhouse. In late September, age 10 weeks, rosette plants were transported to Idaho via overnight shipping. Plants were transplanted directly into the surrounding vegetation in randomized blocks of 50, spaced 10 cm apart. Each block contained 2 plants of each genotype. We planted a total of 800 plants each at JAM and SIL, and 1100 plants at MAH. We watered several times in the following days to establish plants, but they were dependent on natural moisture thereafter. Plants overwintered in the ground, ensuring synchronization with endogenous plants the following spring.

Herbivory and fitness censuses: Several times over the season, we recorded plant traits such as stage, size, top chewed off (TCO), density of the surrounding vegetation, and damage from herbivory. Stages were defined as dead, rosette, bolting, flowers only, flowers and fruits, or fruits only. To quantify vegetation, we estimated the percent plant cover for a 10 cm square centered on the plant. Plants were designated as “TCO” if the entire top of the plant had been removed. Damage was visually assessed as the percentage of tissue removed for rosette leaves, cauline leaves, and fruits; this was done by counting the total number of leaves or fruits, the number that were damaged by herbivores, and the average percent of tissue removed by herbivores on the damaged leaves or fruits. The number damaged was multiplied by the percent of tissue removed, and divided by the total number of leaves/fruits for the final damage number. Damage censuses took place in late July and early August, timed to be as close to peak herbivory as possible while occurring before leaf senescence. Fruit number, adjusted by the percentage of the fruit tissue destroyed by herbivores, was used as a proxy for fitness.

Glucosinolates: We quantified glucosinolates for cauline leaves and immature fruits as in Prasad *et al*. 2012 [21]. Briefly, healthy tissue samples were collected directly into 70% methanol. Tissue collection took place at two time points at each site; at SIL the collections were one day apart, at MAH the collections were separated by seven days, and at JAM the two batches of collections were five days apart. To control for multiple collection times, date of harvest was included as a covariate in analysis of glucosinolate variation. Sample weight was also included as a covariate in some subsequent analyses, as it is correlated with the developmental stages of the fruits. Plant survival and reproduction permitting, leaf and fruit samples were collected from three individuals of each genotype at each site; if there were not enough surviving or reproducing plants of a given genotype, as many samples were collected as possible. Fruits were collected when partially elongated, well before desiccation of fruit tissue or seed maturity. We collected a total of 78 cauline leaves and 58 fruits at JAM, 91 leaves and 91 fruits at MAH, and 99 leaves and 99 fruits at SIL. The reduced sample sizes at JAM were due to high mortality and a number of plants that experienced apical damage before fruit collection.

Samples remained in methanol for 4 weeks to allow leaching of glucosinolates before extraction. The leachate was added to a prepared Sephadex column along with 10 ul of 1mM sinigrin as an internal standard. We washed the columns twice with 70% methanol, twice with water, once with 1 M NaOAC, and twice again with water. Excess liquid was removed by centrifuging columns at 910 G. We added 30 ul of sulfatase and let it stand in the columns overnight. The following morning, samples were eluted in 300 ul of HPLC-grade water and left uncovered overnight before storage at 4 degrees.

We quantified desulfo-glucosinolates in the extracted samples using an Agilent 1100 High-Pressure Liquid Chromatography system, as in Prasad *et al*. 2012 [18]. This was carried out using a ZORBAX Eclipse XDB-C18 4.6 x 150 mm 5-micron column, with a Supelco ColumnSaver 2.0 um filter. The method used HPLC-grade H_2_O and acetonitrile, at a flow rate of 1 ml/minute, at a temperature of 40 degrees. The 30 minute run was: 1.5%–2.5% acetonitrile (6 minutes), 2.5%–5.0% (2 minutes), 5.0%–18.0% (7 minutes), 18.0%–46.0% (2 minutes), 46%–92.0% (6 minutes), 92.0%–1.5% (1 minute), and a hold at 1.5% (6 minutes).

We calculated the values for glucosinolate traits using a custom Python script. The relative response factor (RRF), which corrects for variation in response to UV, for each of these compounds is 1.0, except for 2OH1ME, which has an RRF of 1.32 [28]. The total quantity of each glucosinolate type was calculated as (0.05 micromole sinigrin) * (area of peak for compound x)]/[(area of sinigrin peak) * (RRF)]. We divided the total quantity by the dry weight of the sample to calculate the concentration in micromole/mg.

Total glucosinolate concentration was calculated by summing the concentrations of each compound. Three additional traits, all ratios, were also used. The proportion of branch chain-derived (PropBC) was calculated as the proportion of total glucosinolates that were derived from valine (2OH1ME and 1ME) or isoleucine (2HP). The proportion valine-derived (PropVal) was calculated as the proportion of all branch chain-derived glucosinolates that were derived from valine. Finally, the proportion of 2OH1ME (Prop2OH1ME) was calculated as the proportion of all valine-derived glucosinolates that were 2OH1ME.

### Clipping experiment

We performed a clipping experiment using endogenous plants at three sites, a meadow near JAM, a meadow near MAH, and a similar meadow at 2667 meters elevation (PAR). Tissue removal took place in July at one time point at each site, at a time when most reproductive plants had both flowers and young fruits present. We used four treatments; control (no tissue removed), rosette leaves, cauline leaves, and flowers/fruits. For each of the removal treatments, 1/3 of the tissue was removed, evenly distributed by age; that is, we removed the oldest leaf/fruit, then left the next two and removed the third one, etc. The sample size at each site was 300 plants, with 75 individuals in each treatment. Whole leaves and flowers/fruits were removed. As plants within a location differed in the flowers: fruit ratio when we were collecting, we did not distinguish between the two, but combined them into one treatment where we removed a third of the total number of fruits, flowers and buds. At our sites, most plants had approximately equal numbers of flowers and fruits at the time of removal, although there was variation within populations. Plants were chosen randomly from bolting individuals within the population; treatments were assigned by randomly selecting a label that had a pre-assigned treatment on it. We also recorded height for each plant at the time of tissue removal. String and paper labels were tied to plants and left until the fitness census. We returned to each site 14–26 days later for a fitness census. For all of the plants that we could re-locate, we recorded the number of stalks present, the number of fruits, and the length of the longest fruit. As some labels were lost between removal and censusing, our final sample size was 232 at JAM (55 control, 61 rosette, 55 cauline, and 61 flowers and fruits), 203 at MAH (47 control, 51 rosette, 51 cauline, and 54 flowers and fruits), and 287 plants at PAR (70 control, 74 rosette, 72 cauline, and 71 flowers and fruits), for a final sample size of 722 individuals.

### Statistical analyses

Glucosinolate variation: We performed a multivariate analysis of variance (MANOVA) using JMP 13 (SAS Institute Inc., Cary, NC, USA). The four glucosinolate traits (ConGS, PropBC, PropVal, and Prop2OH1ME) were the response variables, with site, subspecies, sample weight, tissue, stage, harvest date (nested within site and tissue), and the site*tissue, subspecies*tissue, subspecies*site and site*subspecies*tissue interactions as fixed effects and block (nested within site) and genotype (nested within subspecies) as random effects. To improve normality of response variables, PropBC, PropVal, and Prop2OH1ME were arcsine transformed. Block and Genotype were random effects. Sample Weight was included as a proxy for developmental stage. Harvest date was nested within Site and Tissue. When the MANOVA was statistically significant for the Wilk’s Lambda test statistic, subsequent univariate analyses, using Least Squares Restricted Maximum Likelihood models, were used to test the significance of tissue, subspecies, and site on each compound, using a significance threshold of 0.05 [29].

Effects of glucosinolate profile on herbivory: As damage measurements deviated from normality even after transformation, we tested the effects of glucosinolate traits on herbivory using least square means, performed in JMP 13. We used a Standard Least Squares REML model for each tissue, where Trait or Damage = Genotype + Site + Block[Site], with Genotype and Block as random effects, and extracted least square means for glucosinolate traits and damage for each genotype. We then used these least square means to run another model for each tissue, Damage = ConGS_cau + ConGS_fr + PropBC_cau + PropBC_fr + PropVal_cau + PropVal_fr + Prop2OH1ME_cau + Prop2OH1ME_fr. As two models were run independently, we used a Bonferroni correction for multiple tests, resulting in a significance threshold of 0.025.

Fitness effects of tissue damage: The effects of herbivore damage on fitness in the transplant experiment were calculated in R, using a custom script. We used the lme4 package to run a mixed-effects model [30]. Damage variables and fitness were log-transformed to improve normality. The full model was Log(Fruit Number) = Site + Stage + Veg + Log(rosette damage) + Log(cauline damage) + Log(fruit damage) + Block[Site] + Genotype. Block and Genotype were run as random effects. Only individuals that had damage values for all three tissues were included; thus, this analysis only included fruiting individuals, not plants with a seasonal fitness of zero.

In order to compare the slopes of the regression lines for cauline and fruit damage on fruit number, we performed permutation tests of fitness effects in R, using a custom script. Permuted data sets were generated by randomly assigning an individual’s cauline and fruit damage values to one tissue or another, so that an individual was equally likely to end up with the data in the original order, or to have the damage values switched between tissues.

For each permuted data set, we ran the same model as the original, and found the difference between the slope estimates for fruit damage and cauline damage. We ran 2000 permutations to generate a distribution of differences in slope. The *P*-value for the significance of the difference in estimates in the original model was calculated as the proportion of permutation results that were larger than the observed difference.

Effects of clipping treatment on endogenous plant fitness were analyzed using JMP 13. We analyzed the effect of treatment using a Standard Least Squares model, with treatment as our main effect and site, site*treatment, height, and stalk number as covariates. The response, fruit number, was log-transformed to improve normality. Pairwise comparisons between treatments were performed using Tukey HSD.

## Results

### Damage and fitness

**Fig 2:**
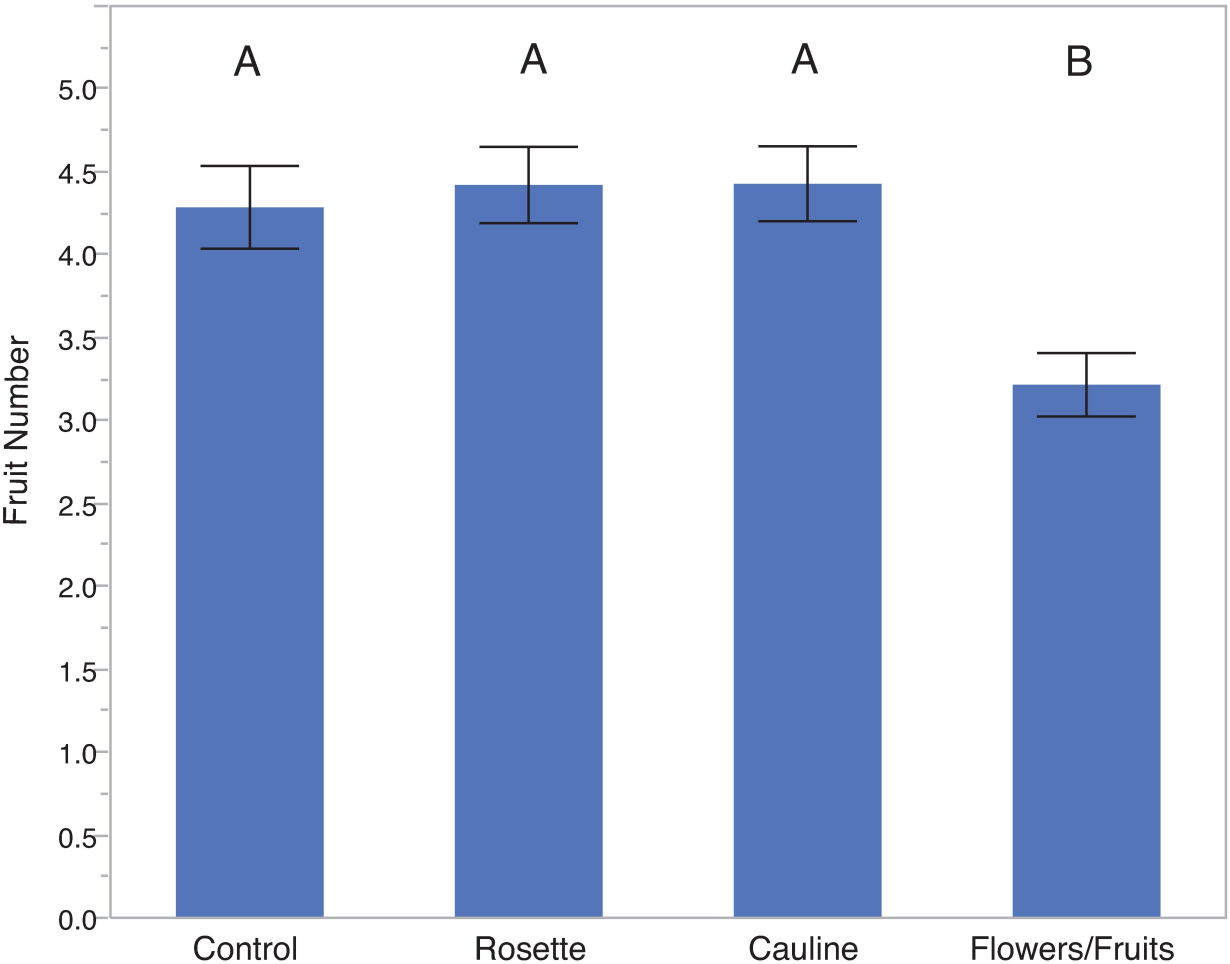
Effects of clipping treatments on fruit number. Endogenous plants were randomly assigned to one of five treatments; for the removal treatments, 1/3^rd^ of the total tissue in question was removed manually. Letters indicate significant pair-wise differences. The control, rosette, and cauline treatments are not significantly different, but flower and fruit removal significantly decreased fruit number. Error bars indicate standard error.

In the clipping experiment, neither the rosette nor the cauline leaf removal treatments reduced fruit number compared to the control treatment. Removal of 33% of the fruits and flowers reduced average fruit number by 30% compared to the control treatment, a statistically significant effect (Table 1; Fig 2).

**Table 1:** Effects of tissue removal treatments on fruit number.

	D.F.	F-ratio	P-value
Treatment	3	24.656	**<0.0001**
Site	2	1.376	0.2530
Treatment*Site	6	0.690	0.6577
Height	1	204.435	**<0.0001**
Stalk Number	1	232.031	**<0.0001**

*N* = 722. Fruit number was log-transformed to improve normality.

**Fig 3:**
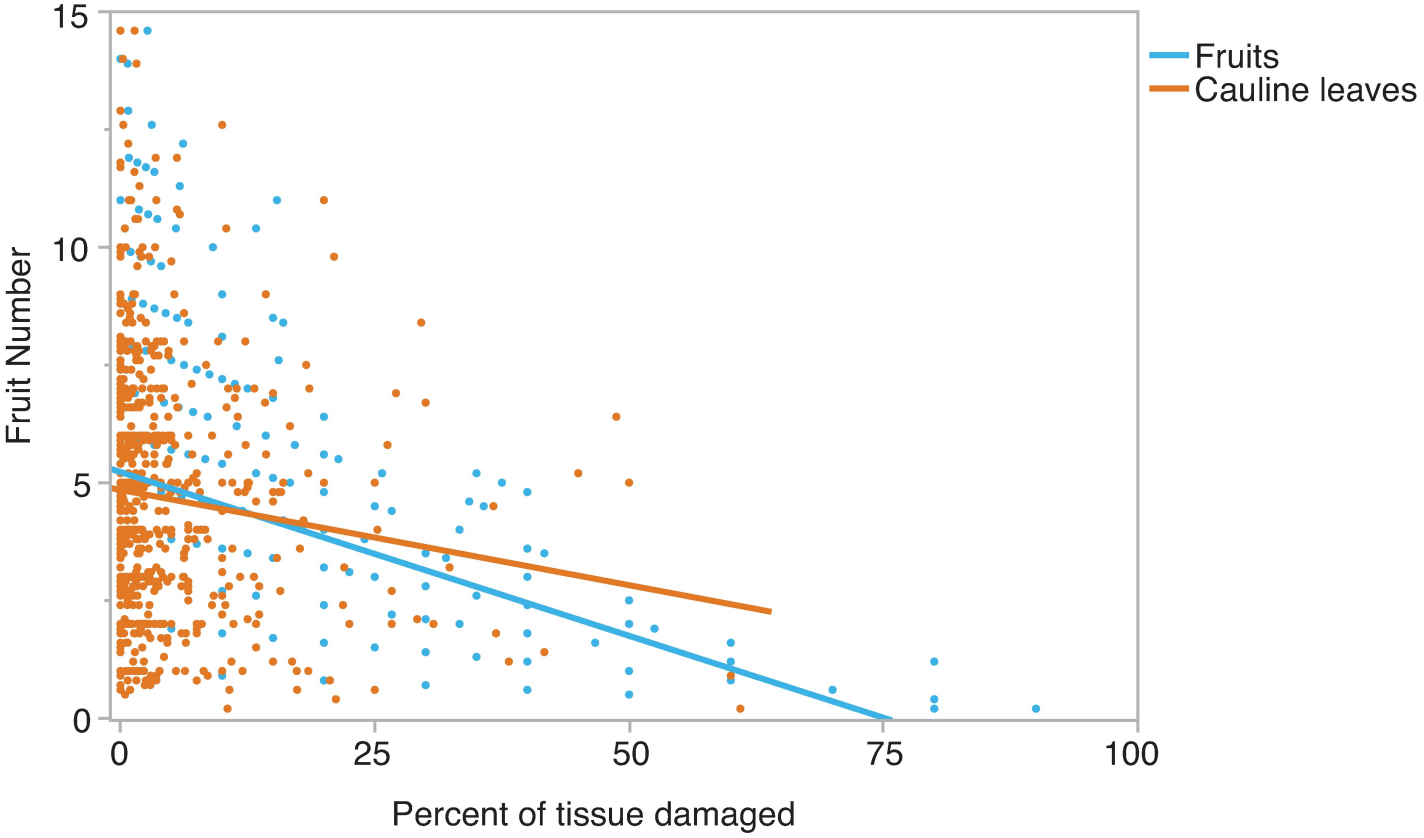
Effects of ambient herbivory on fruit number. Individuals are plotted as points. The slope of the line of fit is equal to the selection gradient on damage to that tissue. Damage to fruits reduces fitness significantly more than loss of cauline leaf tissue (*P*<0.001).

Ambient herbivory removed, on average, 2.7% of rosette leaf tissue, 5.6% of cauline leaf tissue, and 7.2% of fruit tissue. In the transplant experiment, both cauline leaf damage and fruit damage had significant effects on fruit number (Table 2), while rosette leaf damage did not have significant effects in this dataset. Site was also a significant predictor of fitness. The permutation tests indicated that fruit damage had a significantly greater effect on fruit number than cauline damage (Fig 3; *P*<0.001).

**Table 2:** Effects of ambient herbivory on fruit number.

	*P*-value	Estimate
Site	**0.003296**	
Stage	**<0.0001**	
Veg	0.9247	-0.000038
Log(Rosette damage)	0.7496	0.006239
Log(Cauline damage)	**0.0074**	-0.049900
Log(Fruit damage)	**<0.0001**	-0.131700

*P*-values for the effects of tissue damage on plant fitness. *N* = 593. Damage and fitness data were log transformed to improve normality. Estimates are not reported for categorical variables.

### Glucosinolate variation between tissues

**Fig 4:**
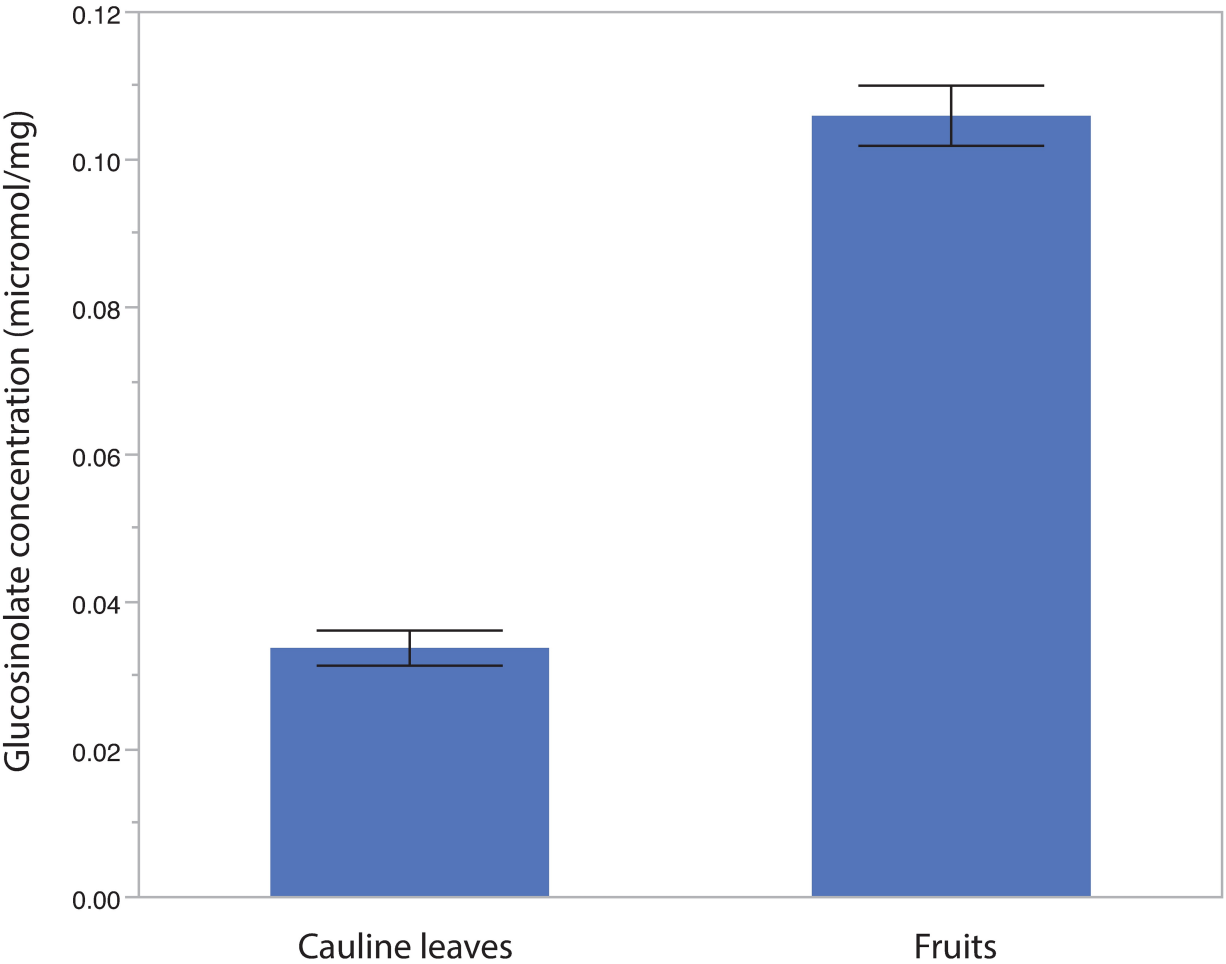
Variation in glucosinolate concentration between cauline leaves and fruits. Fruits have a significantly higher concentration of glucosinolates than cauline leaves do (*P*<0.0001). Error bars indicate standard error.

**Fig 5:**
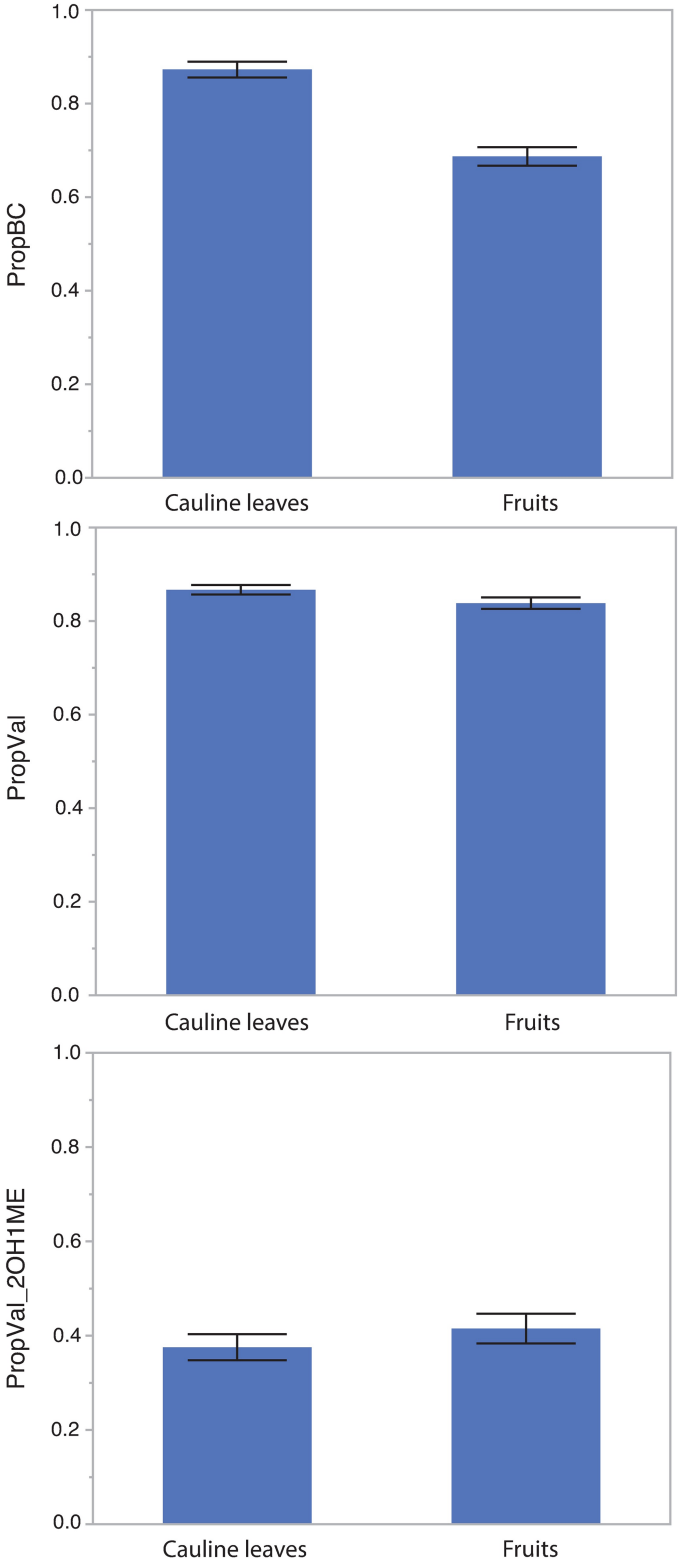
Variation in glucosinolate profile between cauline leaves and fruits. A) The proportion of total glucosinolates that are branch chain-derived glucosinolates, B) The proportion of branch chain-derived that are valine-derived, and C) The proportion of valine-derived that are 2OH1ME. Tissues differ significantly for all three traits. Error bars indicate standard error.

Each sample contained one to four types of glucosinolates. These included one methionine-derived type, 6MSOH, two valine-derived compounds (2OH1ME and 1ME), and one isoleucine-derived compound (2MP). Fruits had, on average, a lower PropBC, lower PropVal, and lower Prop2OH1ME. As the MANOVA model for the effects of tissue, subspecies, and site on glucosinolate traits was significant (*P* < 0.0001), we performed univariate analyses for glucosinolate concentration and each glucosinolate trait. Glucosinolate concentration varied significantly between cauline leaves and fruits (Table 3; Fig 4). On average, glucosinolate concentration was more than 3 times higher in fruits than in cauline leaves. PropBC, PropVal, and Prop2OH1ME also varied significantly between tissues; within-plant variation in glucosinolate profile is common among all traits studied here (Table 3; Fig 5). Sample weight, a proxy for size and developmental state of leaves and fruits, was significant for concentration and PropBC, indicating a potential change in those traits over development.

**Table 3:** Variation in glucosinolate traits.

Factor	Effect type	ConGS N = 485	PropBC N = 483	PropVal N = 448	Prop2OH1ME N = 452
Site	Fixed	0.1294	0.1552	**0.0385**	**0.0029**
Subspecies	Fixed	0.7674	**0.0005**	**<0.0001**	**<0.0001**
Tissue	Fixed	**<0.0001**	**<0.0001**	**0.0132**	**0.0174**
Stage	Fixed	**0.0009**	0.1695	**0.0435**	0.1988
Sample weight	Fixed	**<0.0001**	**<0.0001**	0.0701	0.6449
Harvest date	Fixed	0.4204	0.3120	0.9859	0.7378
Site*Tissue	Fixed	0.1285	**<0.0001**	0.1516	0.5338
Subspecies*Tissue	Fixed	0.9235	**0.0227**	**0.0001**	**<0.0001**
Subspecies*site	Fixed	**0.0189**	0.0934	0.0574	<**0.0001**
Subsp*Site*Tissue	Fixed	0.8558	**0.0362**	0.7455	0.4022
Genotype[Subspecies]	Random	0.1697	**0.0015**	**0.0047**	0.0565
Block[Site]	Random	0.1463	0.2098	0.3069	0.9313

*P-*values for variation in glucosinolate traits. Prop_BC is calculated as proportion of total glucosinolates derived from branch chain amino acids (BC-derived). PropVal is the proportion of BC-derived glucosinolates that are valine-derived, and Prop2OH1ME is the proportion of valine-derived glucosinolates that are made into 2OH1ME. PropBC, PropVal and Prop2OH1ME were arcsine transformed to improve normality. Statistically significant effects (*P* < 0.05) are indicated in bold.

We also found variation in glucosinolate profile between subspecies for all traits except concentration (S1 Fig), and among genotypes within subspecies for PropBC and PropVal. There was a significant Subspecies*Tissue interaction for PropBC, PropVal and Prop2OH1ME, but not for concentration (S2 Fig). Allocation of total glucosinolates between leaves and fruits does not differ between subspecies, although other components of the glucosinolate profile do.

### Effects of glucosinolate profile on herbivory

After correcting for multiple tests, no glucosinolate traits were significant predictors of leaf or fruit damage in the field (Table 4).

**Table 4:** Effects of glucosinolate traits on tissue damage.

	Cauline damage	Fruit damage
ConGS_cauline	0.5276	0.6530
PropBC_cauline	0.1916	0.1145
PropVal_cauline	0.8261	0.9683
Prop2OH1ME_cauline	0.1295	0.0962
ConGS_fruit	0.2327	0.9727
PropBC_fruit	0.7595	0.7739
PropVal_fruit	0.3139	0.3201
Prop2OH1ME_fruit	0.1254	0.1240

*P-*values for effects of glucosinolate traits on damage to cauline leaves and fruits. Due to deviations from normality for the individual data, these were calculated using genotype least square means (n = 24). Using a Bonferroni correction for 2 tests, the significance threshold is 0.025.

## Discussion

Our results support the ODH. In both the clipping treatment and the ambient herbivory experiment, we found that damage to fruits was significantly more detrimental to fitness than loss of leaf tissue. Correspondingly, fruits had a much higher concentration of glucosinolates. These results support the commonly-made assumption that reproductive tissues are of higher fitness value than vegetative tissues. These results also unambiguously support the ODH; the tissue of higher value has a higher investment in defense.

Loss of leaf tissue was not necessarily costly to fitness. We found that loss of rosette tissue, from either clipping or ambient herbivory, had no detectable costs to fitness, in contrast to previous evidence in *B*. *stricta* [21]. This may be because this analysis considered only fruiting plants; the impacts of rosette damage may be greater in earlier developmental stages, such as in determining whether a plant bolts in a given year or not. The clipping experiment also found that removing cauline tissue had no effect on fruit number, a result which differed from the ambient herbivory experiment. This difference could be due to clipping failing to accurately mimic the effects of herbivory. Our clipping method, which removed whole leaves, is not identical to herbivory, which is more likely to remove only part of a leaf, generally along the margins. If the costs of damage are due to effects other than simply the loss of photosynthetic tissue, clipping may not accurately simulate herbivory. Cauline leaf tissue may also simply not have high value by the time that plants begin flowering. Given, however, that the ambient herbivory experiment did not have high levels of observable herbivory prior to flowering, we do not think that this explanation is the most likely. Actual herbivory on leaves was also less detrimental than herbivory on flowers, the same pattern as observed in the manipulated plants; thus, even if clipping is not a perfect mimic of herbivory on leaves, we find consistent evidence that fruits are more valuable than leaves.

We did not observe compensation for clipping of reproductive tissue; removal of approximately 30% of flowers and young fruits resulted in a proportional reduction in final fruit number. Multiple mechanisms of compensation for flower loss have been described, including development of additional buds, reduced abortion of fruits, or maturation of “reserve” flowers or ovules that would not otherwise produce seeds [12, 13]. The latter was the mechanism we hypothesized to act in *B*. *stricta*, as we often observe buds that do not develop into flowers or fruits. Such compensation was not evident, however. The ability of a plant to compensate for floral loss may depend on the timing of the damage [13]. *B*. *stricta* might be able to compensate more effectively for reproductive damage that occurred earlier in the season; however, our clipping experiment was timed to coincide with the peak activity of herbivores that we observe. Thus, the data from our clipping experiment did not support the hypothesis that *B*. *stricta* is able to compensate for the loss of young reproductive tissues. The lack of compensation indicates that these tissues do have very high fitness value, consistent with the high levels of defenses.

Our results support the assumption that reproductive tissues have higher fitness value than leaves. Given contradictory results from studies of floral value, however [1, 10], the question is not entirely resolved. The discrepancies in the results may be due to differences in value between flowers and fruits, even when immature; we had no clipping treatment that was exclusively removing flowers, and it was logistically infeasible to quantify ambient florivory in the transplant experiment. Any generalizations about the relative values and defenses of leaves, flowers and fruits must consider the mutualistic interactions with insects, as well as herbivory; attracting pollinators and dispersers may also play a role in shaping defenses, along with deterring herbivores [19, 20]. In addition, the value of flowers may vary widely between species that self-pollinate, where almost every flower might be expected to set seed, and outcrossers, where floral success is not guaranteed, and protecting floral tissues may affect pollination potential. Here, we eliminate significant mutualisms by using a self-pollinating, self-dispersing species; without these mutualisms, the ODH holds true. More work is needed, however, to clarify whether this is true in other mating and dispersal systems.

Glucosinolate traits other than concentration also vary between tissues; all three of the proportional traits varied significantly between cauline leaves and fruits. Similar tissue-specific variation in glucosinolate profile has been found in Arabidopsis, where reproductive tissues have a higher diversity of glucosinolates than vegetative parts, including types that are not found elsewhere in the plant [17]. Tissue-specific patterns have also been found in other Brassicaceae species [31]. Within-plant variation in glucosinolate types appears to be common. Such variation could be due to differing herbivore pressures on each tissue; if the herbivore community varies, so too could selection pressures on glucosinolates [32]. Our results, however, do not provide strong support this hypothesis; no glucosinolate traits were significant in predicting herbivory on either tissue. Glucosinolate profile has been previously demonstrated to affect leaf herbivory; however, detection of such effects has required very large sample sizes [21]. This study may simply have lacked the power to find significant effects. Overall, these data do not provide evidence that the effects of glucosinolate profile on herbivory vary between tissues. Alternative hypotheses include pleiotropic or developmental effects. In *A*. *thaliana*, glucosinolates are transported within the plant with a high degree of specificity of type [33]; if transporters preferentially move specific compounds, that could also cause within-plant variation. Studies of the effect of glucosinolates on herbivory in other crucifer species have had complex results. Some have found clear effects of glucosinolate profile on damage or herbivore community composition [34–36], although such effects have not been universal [37]. Numerous studies have found that the importance and effect of glucosinolate profile depends on the herbivore species [32, 36, 38–42]. Here, we cannot rule out herbivory as a factor leading to glucosinolate variation within the plant, but neither do our data support it as a hypothesis.

Previous work in *B*. *stricta* has demonstrated that genetic variation in glucosinolates is under selection, and may be important in determining the extent of herbivore damage to the plant [21]. Here, we find that glucosinolates vary on several scales; between subspecies, among genotypes within a subspecies, and among tissues within an individual plant. We find that this within-plant variation in glucosinolate concentration is consistent with the adaptive explanation of the ODH, with a greater investment of defenses in the more valuable tissues of the plant.

## Supporting information

S1 Fig Differences in glucosinolate concentration and profile between subspecies. Differences were significant for PropBC, PropVal, and PropVal_2OH1ME (*P* < 0.0125). Error bars represent standard error.
(TIF)

S2 Fig Subspecies * Tissue interactions for glucosinolate concentration and profile. The interaction effect was significant for PropVal and PropVal_2OH1ME (*P* < 0.0125).
(TIF)

S1 File A Python script to parse output data from HPLC results. (PY)

S2 File A Python script to merge glucosinolate data with a metafile of plant data. (PY)

S3 File A Python script for calculations of glucosinolate traits. (PY)

S4 File An R script to calculate the effects of herbivory on fitness, and perform a permutation test comparing the effects of cauline leaf damage and fruit damage. (R)
