# Supplementary figures and images for: Testing the optimal defense hypothesis in nature: Variation for glucosinolate profiles within plants
Source: PLoS One. 2017 Jul 21;12(7):e0180971. doi: 10.1371/journal.pone.0180971 (PMC5521783; doi:10.1371/journal.pone.0180971)

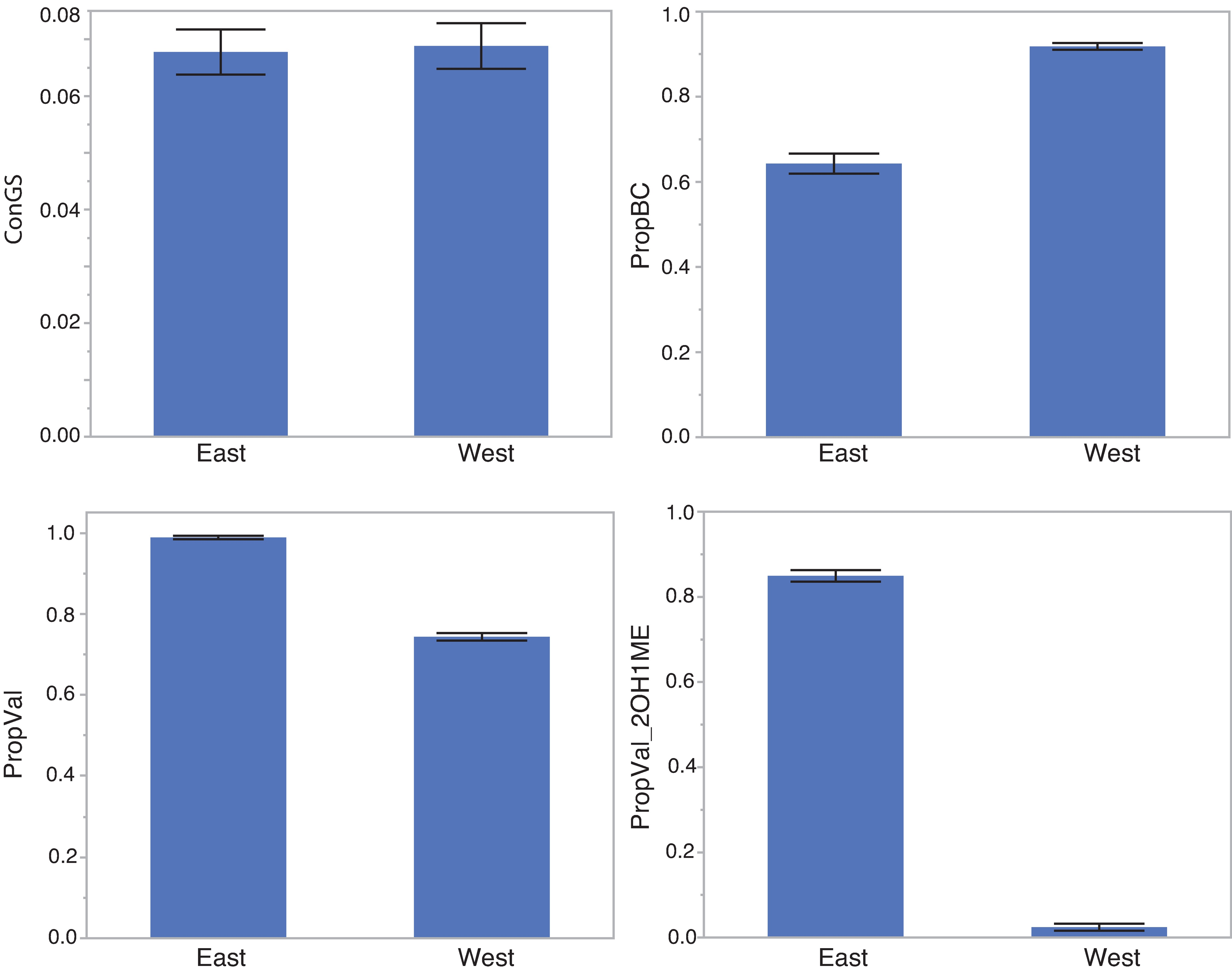

Supplement: S1 Fig — Differences were significant for PropBC, PropVal, and PropVal_2OH1ME (P < 0.0125). Error bars represent standard error. (TIF) [file pone.0180971.s001.tif]

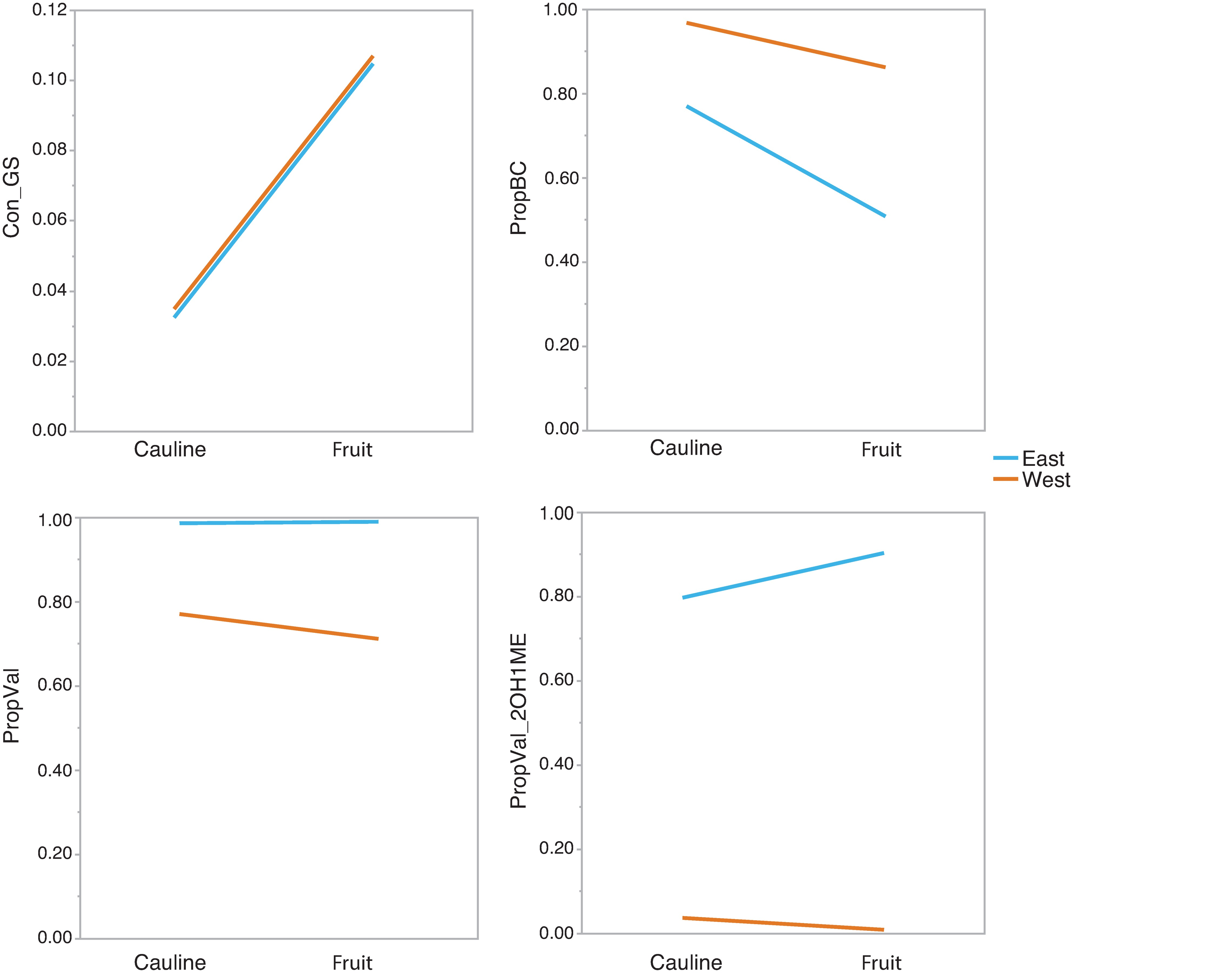

Supplement: S2 Fig — The interaction effect was significant for PropVal and PropVal_2OH1ME (P < 0.0125). (TIF) [file pone.0180971.s002.tif]
